# The Multifaceted Role of Autophagy in Nasopharyngeal Carcinoma: Translational Perspectives on Pathogenesis, Biomarkers, Treatment Resistance, and Emerging Therapies

**DOI:** 10.3390/cancers17152577

**Published:** 2025-08-05

**Authors:** Abdul L. Shakerdi, Emma Finnegan, Yin-Yin Sheng, Graham P. Pidgeon

**Affiliations:** Department of Surgery, Trinity Translational Medicine Institute, St James’s Hospital & Trinity College Dublin, D08 NHY1 Dublin, Ireland; shakerda@tcd.ie (A.L.S.); finnege3@tcd.ie (E.F.); shengyi@tcd.ie (Y.-Y.S.)

**Keywords:** nasopharyngeal carcinoma, autophagy, Epstein–Barr virus, therapeutic resistance, biomarkers, targeted therapy, translational research

## Abstract

**Background:** Nasopharyngeal carcinoma (NPC) is an epithelial malignancy arising from the nasopharyngeal mucosa. Despite treatment advances such as the use of intensity-modulated radiotherapy and immune checkpoint inhibitors, resistance remains a significant clinical challenge. Many tumours are also diagnosed at an advanced stage associated with poor prognosis. **Objective:** This review aims to explore the biological roles of autophagy in NPC, primarily highlighting its involvement in disease pathogenesis and treatment resistance. **Methods:** We performed a review of the recent literature examining the role of autophagy-related pathways in NPC pathogenesis, biomarker discovery, and therapeutic targeting. **Results:** Autophagy plays a dual role in NPC as it contributes to both tumour suppression and progression. It is involved in tumour initiation, metastasis, immune modulation, and treatment resistance. Autophagy-related genes such as SQSTM1, Beclin-1, and AURKA may serve as prognostic and therapeutic biomarkers. Various strategies are being investigated for their role to modulate autophagy using pharmacologic inhibitors, RNA interventions, and natural compounds. **Conclusions:** Further research into autophagy’s context-dependent roles in NPC may inform the development of personalised therapies and allow progress in translational and precision oncology.

## Simple Summary

Nasopharyngeal carcinoma (NPC) is a type of cancer that forms in the upper part of the throat, behind the nose. Although rare in most parts of the world, it is much more common in certain regions such as Southeast Asia. This review focuses on a process called autophagy, a natural mechanism cells use to break down and recycle their components. In NPC, autophagy plays a complex role. It can help cancer cells survive under stress such as chemotherapy, but it can also trigger cell death under certain conditions. Understanding how autophagy is regulated in NPC may reveal new ways to improve treatment outcomes. We summarise the current knowledge about autophagy’s dual role in NPC progression and resistance to therapy and explore potential strategies to target autophagy in future treatments. This article is intended to help researchers and clinicians better understand how manipulating autophagy could contribute to more effective NPC therapies.

## 1. Introduction

Nasopharyngeal carcinoma (NPC) is an epithelial malignancy originating from the nasopharyngeal mucosa, most commonly the lateral pharyngeal recess [1]. Although NPC is considered a relatively rare cancer globally, it demonstrates a marked geographic and ethnic distribution, with high incidence rates in Southern China, Southeast Asia, North Africa, and Inuit populations of North America [2]. The age-standardised annual incidence of NPC in endemic regions ranges from approximately 3 to 15 per 100,000 people, compared to fewer than 1 in 100,000 in non-endemic Western populations [3]. Histopathologically, NPC is classified according to the World Health Organisation (WHO) into three distinct subtypes: keratinising squamous cell carcinoma (WHO type I), non-keratinising carcinoma (WHO type II), and undifferentiated carcinoma (WHO type III) [4]. A distinguishing feature of NPC, compared to other head and neck squamous cell carcinomas (HNSCCs), is its strong association with Epstein–Barr virus (EBV) infection [5]. In endemic regions, EBV DNA is detectable in virtually all cases of non-keratinising and undifferentiated subtypes [6]. EBV positivity in keratinising subtypes is variable, and it is frequently positive in endemic areas for NPC but rarely present in low-incidence areas [7,8,9]. Notably, non-keratinising tumours are associated with more favourable survival outcomes than keratinising tumours [10]. Proposed mechanisms to justify the decreased overall survival in the keratinising subtype include the decreased immune response associated with lower EBV positivity and increased chemoradiation resistance in this phenotype [11,12,13]. In addition to viral oncogenesis, a range of other environmental and genetic risk factors have been implicated in NPC pathogenesis, including the consumption of salted fish and other nitrosamine-rich preserved foods, tobacco consumption, alcohol intake, chronic sinonasal inflammation, and inherited susceptibility loci [14,15,16]. Despite advances in the early detection and treatment of NPC, a significant proportion (approximately 70%) of cases are diagnosed at an advanced stages and are therefore associated with poorer clinical outcomes [17]. 

The current standard of care for regionally advanced NPC often involves radiotherapy in the form of intensity-modulated radiotherapy (IMRT), with or without platinum-based chemotherapy, across all subtypes [18]. Recent studies have highlighted the potential benefit of induction chemotherapy prior to concurrent chemoradiation (CCRT), as evidence shows it can improve clinical outcomes when compared with CCRT alone [19]. Recurrent and metastatic NPC has historically been associated with poor outcomes, but immune checkpoint inhibitors (ICIs) such as nivolumab and camrelizumab (anti-PD-1) have recently shown promise with objective response rates of 20% and 34%, respectively, as monotherapies [20,21]. Despite these advances, resistance to radiotherapy, chemotherapy, and immunotherapy continues to pose a significant clinical challenge, highlighting the need for a better understanding of the pathophysiology of NPC in order to develop future therapies and enable the identification of novel biomarkers for earlier diagnosis.

Autophagy is an evolutionarily conserved catabolic process by which cells degrade and recycle cytoplasmic constituents in response to stressors such as nutrient deprivation or hypoxia [22]. During autophagy, cytosolic constituents are sequestered within double-membraned vesicles called autophagosomes which subsequently fuse with lysosomes to enable enzymatic degradation of the cargo. This multistep process can be broadly divided into four phases: initiation, nucleation, expansion/elongation, and fusion and degradation [23]. Under basal conditions, autophagy serves as a crucial quality control and homeostatic mechanism. Autophagy is tightly regulated by nutrient-sensing pathways, particularly the AMP-activated protein kinase (AMPK)/mammalian target of rapamycin (mTOR) axis, along with a suite of autophagy-related (Atg) proteins. Activation of autophagy is initiated upon inhibition of the mTORC1 complex, which consists of mTOR associated with proteins like proline-rich Akt substrate of 40 kDa (PRAS40) and regulatory-associated protein of mTOR (Raptor) [24]. This results in the dephosphorylation of Atg13, permitting its interaction with and activation of the UNC-51-like kinase 1 (ULK1) complex [25]. Focal adhesion kinase family-interacting protein of 200 kD (FIP200) acts as an essential cofactor in this process by facilitating the assembly of downstream autophagic machinery. This activation marks the initiation phase, leading to the formation of a membrane structure called the phagophore, which serves as the precursor to the autophagosome [26,27]. The formation of the autophagosomal bilayer relies on the activity of phosphatidylinositol kinase P150 and Beclin-1. During the nucleation phase, class III PI3K complex I, including proteins like vacuolar protein sorting 34 (VPS34), VPS15, mAtg13, nuclear receptor-binding factor 2 (NRBF2), and Beclin-1, promotes phosphatidylinositol 3-phosphate (PI3P) production [28,29]. LC3, a ubiquitin-like autophagy-related protein, is lipidated by conjugation to phosphatidylethanolamine to form LC3-II, which subsequently integrates into autophagosomal membranes [30]. Following autophagosome formation, the fusion and degradation phase begins. During this phase, the autophagosome fuses with lysosomes, forming an autolysosome, where the enclosed cargo is broken down by lysosomal hydrolases [31]. Class III PI3K complex II, composed of proteins such as VPS15, VPS34, UV radiation resistance-associated gene (UVRAG), and Beclin-1, is believed to play a role in autophagosomal maturation and trafficking during this stage [32,33]. Autophagy can also occur in a selective manner, where specific damaged organelles, protein aggregates, or intracellular pathogens are targeted for removal [34]. Selective autophagy is facilitated by autophagy cargo receptors such as p62/sequestosome-1 (SQSTM1), which bind to ubiquitylated cargo and interact with LC3-II, directing the cargo into the forming autophagosome [35]. Autophagy has recently emerged as a regulator of tumour initiation, progression, and therapeutic resistance in NPC. Therefore, it offers significant promise in the identification of novel biomarkers and therapeutic targets, which may improve disease outcomes through earlier diagnosis and enhanced therapeutic efficacy. 

## 2. Autophagy in the Initiation and Progression of Nasopharyngeal Carcinoma

Autophagy plays a complex, context-dependent role in cancer biology, functioning as both a tumour-suppressive and tumour-promoting process [36]. In the early stages of tumourigenesis, autophagy can prevent malignant transformation by removing dysfunctional mitochondria and protein aggregates, thereby limiting oxidative stress, genomic instability, and aberrant oncogenic signalling [37]. In NPC, several lines of evidence support a tumour-suppressive role for autophagy. Zhao and colleagues demonstrated that expression of WD repeat domain phosphoinositide-interacting protein 1 (WIPI-1) was reduced in NPC cells and tumour tissues [24]. WIPI-1, which interacts with the E3 ubiquitin-protein ligase TRIM21 to enhance autophagic flux, was found to inhibit NPC cell proliferation and migration in vitro and suppress metastasis to popliteal lymph nodes and lungs in vivo. Low WIPI-1 expression also was significantly associated with poorer progression-free survival (PFS) in NPC patients [38]. Subsequent research has shown that Pin2 telomeric repeat factor 1-interacting telomerase inhibitor 1 (PinX1) expression was decreased in the CD133+ cancer stem [39]. It has since been demonstrated that PinX1 promotes autophagy by inhibiting the AKT/mTOR pathway to exert tumour-suppressive effects in NPC [40]. In addition, tumour necrosis factor-α–induced protein 8 like-1 (TIPE1) is overexpressed in NPC tissues and correlates with the proliferation marker Ki67 [41]. TIPE1 reduces autophagy through interaction with the AMPK/mTOR signalling pathway, and its expression in NPC tissues was significantly associated with shorter overall survival (OS) [41].

**Figure 1:**
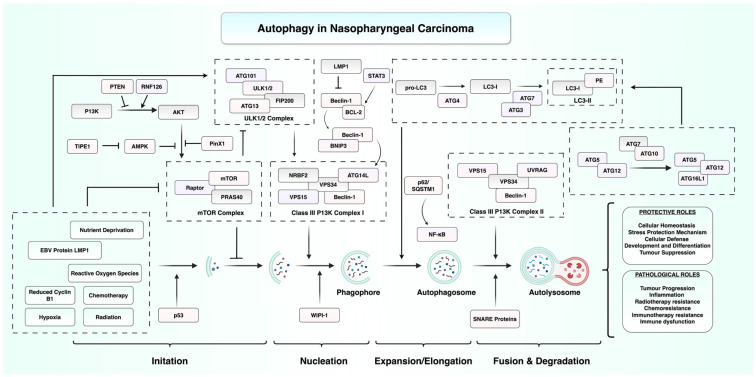
**Overview of autophagy regulation in NPC**. This diagram illustrates the multistep autophagy process from initiation, nucleation, expansion/elongation, to fusion and degradation. It highlights the complex interplay between host signalling pathways and EBV-associated factors. EBV latent membrane protein 1 (LMP1) and other cellular stressors modulate autophagy through inhibition of the phosphoinositide 3-kinase (PI3K)/AKT/mammalian target of rapamycin (mTOR) axis or activation of AMP-activated protein kinase (AMPK). These signals regulate autophagy-related complexes and proteins such as the ULK1/2 complex, Beclin-1, and LC3, ultimately leading to the formation of autophagosomes and their fusion with lysosomes to generate autolysosomes. Abbreviations: AKT, protein kinase B; AMPK, AMP-activated protein kinase; ATG, autophagy-related gene; BCL-2, B cell lymphoma 2; BNIP3, BCL2-interacting protein 3; EBV, Epstein–Barr virus; LC3, microtubule-associated protein 1A/1B-light chain 3; LMP1, latent membrane protein 1; mTOR, mammalian target of rapamycin; NF-κB, nuclear factor kappa-light-chain-enhancer of activated B cells; NPC, nasopharyngeal carcinoma; PE, phosphatidylethanolamine; PI3K, phosphoinositide 3-kinase; PINK1, PTEN-induced putative kinase 1; PRAS40, proline-rich AKT substrate of 40 kDa; PTEN, phosphatase and tensin homolog; RNF126, ring finger protein 126; SNARE, soluble NSF attachment protein receptor; STAT3, signal transducer and activator of transcription 3; SQSTM1/p62, sequestosome 1; TFE1, transcription factor E1; ULK1/2, Unc-51-like kinase 1/2; UVRAG, UV radiation resistance-associated gene; VPS, vacuolar protein sorting; WIPI-1, WD repeat domain phosphoinositide-interacting protein 1. Figure created using BioRender.com (URL accessed on 31 July 2025) and Microsoft PowerPoint.

In contrast to discussed evidence of autophagy acting as a tumour suppressor, accumulating evidence highlights its pro-tumourigenic role in NPC. Xie et al. reported that knockdown of cyclin B1 induced autophagy in CNE1 and CNE2 NPC cell lines, by increasing the level of reactive oxygen species (ROS), resulting in the activation of the AMPK-ULK1-dependent signalling pathway [42]. Notably, this activation of autophagy may demonstrate a cytoprotective response, suggesting that under stressful conditions like cyclin B1 depletion, NPC cells may exploit autophagy to enhance their survival. Further supporting the pro-tumourigenic role of autophagy, a recent study has demonstrated that overexpression of Wnt5a in NPC cells enhances autophagosome formation and upregulates key autophagy markers, including Beclin1 and LC3B [43]. This activation contributes to radioresistance in NPC [43], suggesting that autophagy may be exploited by cancer cells in response to therapeutic stress. In addition, accumulation of the autophagy adaptor protein p62/SQSTM1 has been shown to drive epithelial–mesenchymal transition (EMT) through the NF-κB pathway, promoting metastasis. The inhibition of p62 expression sensitises NPC cells to cisplatin chemotherapy [44]. Figure 1 summarises the role of the autophagy pathways in NPC. 

### 2.1. EBV Infection and Autophagy 

EBV is a double-stranded DNA virus belonging to the gammaherpesvirus family, characterised by its ability to establish infections in human B lymphocytes and epithelial cells [45,46]. The virus is classified as a Group 1 carcinogen by the International Agency for Research on Cancer (IARC) due to its association with several cancers including NPC, Burkitt’s lymphoma, Hodgkin’s lymphoma, and some cases of gastric cancer [47]. In NPC, EBV persists in a latent state, characterised by the expression of specific viral genes including latent membrane proteins (LMP1, LMP2A/B), EBV nuclear antigens (EBNA1), abundant non-coding RNAs like EBV-encoded small RNAs (EBERs), and BamHI-A rightward transcript (BART) microRNAs [48,49]. These viral products modulate host signalling pathways, inhibit apoptosis, promote immune evasion, and are involved in metabolic reprogramming. 

**Figure 2:**
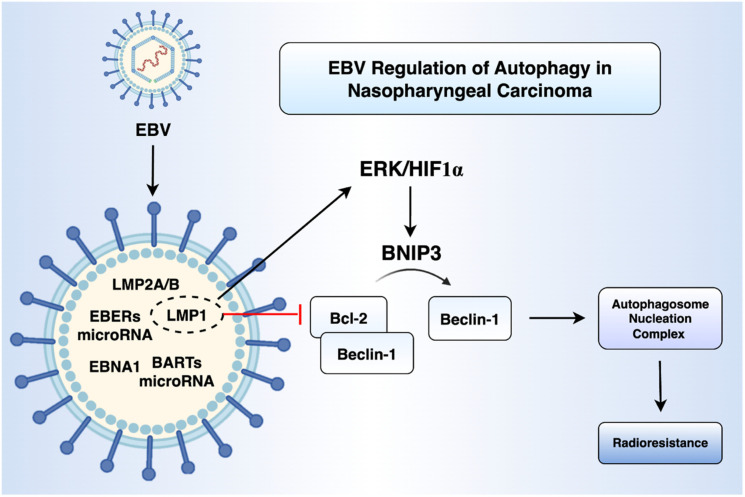
**Schematic representation of EBV-mediated regulation of autophagy in NPC.** EBV expresses a range of latent genes, including latent membrane proteins 1 and 2A/B (LMP1, LMP2A/B), EBV-encoded small RNAs (EBERs), Epstein–Barr nuclear antigen 1 (EBNA1), and BamHI-A rightward transcript microRNAs (BART miRNAs). Among these, LMP1 activates the extracellular signal-regulated kinase (ERK) and hypoxia-inducible factor 1-alpha (HIF1α) signalling pathway, leading to upregulation of BCL2-interacting protein 3 (BNIP3). BNIP3 disrupts the inhibitory B cell lymphoma 2 (Bcl-2)–Beclin-1 complex, releasing Beclin-1 to promote the formation of the autophagosome nucleation complex. This EBV-driven autophagy contributes to enhanced radioresistance in NPC cells. Figure created using BioRender.com (URL accessed on 31 July 2025) and Microsoft PowerPoint.

It has recently been demonstrated that LMP1 promotes autophagy by upregulating the expression of BNIP3 through the ERK/HIF1α signalling axis (Figure 2). BNIP3 disrupts the Bcl-2-Beclin-1 complex, freeing Beclin-1 to assemble the autophagosome nucleation complex. LMP1 also promotes the binding of BNIP3 to Beclin-1 and inhibits the binding of Bcl-2 to Beclin1 [50]. Functionally, this LMP1-mediated autophagy has been implicated in radioresistance. Furthermore, knockdown of BNIP3 can sensitise LMP1-positive NPC cells to radiation both in vitro and in vivo [50]. Yiu et al. further demonstrated that autophagy is essential for the reactivation of EBV’s lytic cycle by the iron chelator C7 in NPC cells [51]. In their studies, pharmacological inhibition of autophagy initiation with 3-methyladenine abolished C7-induced expression of the EBV immediate-early protein Zta, whereas blockade of autolysosome formation with chloroquine had no effect. Systematic siRNA silencing of key autophagy-related genes revealed that only depletion of ATG5 prevented Zta induction across three EBV-positive cell lines. These findings support a dual-inducer strategy combining lytic reactivation and autophagy modulation as a potential therapeutic approach for EBV-associated malignancies.

### 2.2. RNA-Mediated and Epigenetic Control of Autophagy in NPC

Epigenetic dysregulation has been increasingly recognised as a driver of tumour initiation and progression. Long non-coding RNAs (lncRNAs) are defined as transcripts >200 nucleotides in length with no protein-coding potential that play a crucial role in modulating gene expression [52]. They work primarily by recruiting chromatin-modifying complexes to specific genomic loci, thereby regulating DNA methylation and histone modifications [52]. LncRNAs have been increasingly recognised as regulators of autophagy in NPC. LINC00324, for example, serves as a sponge for miR-3164, preventing repression of peptidylarginine deiminase 4 (PAD4), which activates the PI3k/Akt pathway to suppress autophagy and apoptosis [53]. 

Another lncRNA, LINC00313, is highly expressed in NPC and has been recently implicated in maintaining stemness and repressing autophagy. Xu et al. reported that silencing of LINC00313 drives an increase in autophagic flux and reduced stem-like properties [54]. Mechanistically, LINC00313 binds the RNA-binding protein PTBP1, forming a complex that upregulates STIM1, which in turn suppresses autophagy through the Akt/mTOR pathway. Interestingly, LINC00313 is also modified post-transcriptionally via N6-methyladenosine (m6A) methylation, installed by the methyltransferase METTL3, and recognised by the reader protein IGF2BP1, highlighting a layer of epi-transcriptional regulation [40]. In contrast, ZFAS1 is an lncRNA that promotes autophagy by sequestering miR-100-3p, hence relieving repression of ATG10 [55]. Stability of ZFAS1 is also under regulation of the m6A methyltransferase METTL3. Functionally, ZFAS1 enhances NPC cell proliferation, migration (transwell assays), and EMT, as evidenced by increased expression of the mesenchymal markers (*N*-cadherin and vimentin) and reduced expression of E-Cadherin [55]. 

**Figure 3:**
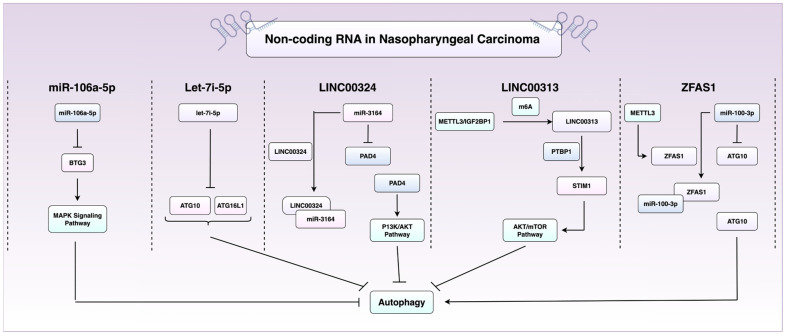
**Regulation of autophagy by non-coding RNAs in NPC.** This schematic summarises key non-coding RNAs that influence autophagy in NPC. Long non-coding RNAs (lncRNAs) such as LINC00324 and LINC00313 inhibit autophagy through activation of the PI3K/AKT and AKT/mTOR signalling pathways, respectively. LINC00324 acts via the miR-3164/PAD4 axis, while LINC00313 functions through PTBP1/STIM1. MicroRNAs let-7i-5p and miR-106a-5p also suppress autophagy by targeting autophagy-related genes (ATG10, ATG16L1) and BTG3. In contrast, the lncRNA ZFAS1 promotes autophagy by sponging miR-100-3p, thereby upregulating ATG10. Abbreviations: AKT, protein kinase B; ATG, autophagy-related gene; BTG3, B cell translocation gene 3; METTL3, methyltransferase-like 3; miR, microRNA; mTOR, mammalian target of rapamycin; NPC, nasopharyngeal carcinoma; PAD4, peptidylarginine deiminase 4; PI3K, phosphoinositide 3-kinase; PTBP1, polypyrimidine tract-binding protein 1; STIM1, stromal interaction molecule 1; ZFAS1, zinc finger antisense 1. Figure created using BioRender.com (URL accessed on 31 July 2025) and Microsoft PowerPoint.

MicroRNAs also regulate autophagy and contribute to NPC malignancy. Two oncomiRs, let-7i-5p and miR-106a-5p, are microRNAs that have been found to drive malignant phenotypes in NPC by functioning as autophagy suppressors [42]. You et al. demonstrated that let-7i-5p targets ATG10 and ATG16L1 in NPC to suppress autophagy and confer a malignant phenotype. Moreover, they found using a cohort of 150 NPC tissues that let-7i-5p levels positively correlated with an advanced stage, metastasis, recurrence, and poorer clinical outcomes [56]. Similarly, miR-106a-5p functions as an autophagy suppressor by targeting BTG3 and activating MAPK signalling, and it is also associated with an advanced stage, metastasis, and poor outcomes [57]. Collectively, these studies highlight the essential role of non-coding RNAs in the epigenetic and post-transcriptional regulation of autophagy in NPC (Figure 3) and emphasise their potential as therapeutic or prognostic biomarkers.

### 2.3. Immune Regulation by Autophagy

Autophagy has also been shown to play a critical role in shaping the tumoural immune microenvironment. A recent study by Yu et al. demonstrated that NPC-derived exosomes enriched with the E3 ubiquitin ligase RNF126 promote tumour progression by altering the tumour immune microenvironment [58]. Mechanistically, RNF126 was shown to induce the ubiquitination and degradation of PTEN in macrophages, leading to activation of the PI3K/AKT signalling pathway and suppression of autophagy. This suppression of autophagy promotes the polarisation of macrophages toward an M2 phenotype, which is associated with tumour progression and immune evasion. Importantly, pharmacological activation of autophagy with rapamycin reversed the RNF126-induced M2 polarisation [58]. These findings indicate that modulation of the RNF126–PTEN–PI3K/AKT–autophagy axis may represent a novel strategy for immunomodulatory therapy in NPC.

## 3. Therapeutic Resistance: The Role of Autophagy

### 3.1. Radioresistance

Both intrinsic and acquired resistance to radiotherapy pose significant clinical challenges in the treatment of NPC. A growing body of evidence suggests that NPC cells can evade radiation-induced death by modulating autophagy through several molecular pathways. One major route is via suppression of PI3k/Akt/mTOR signalling. Annexin A6 (ANXA6) is a calcium-dependent phospholipid-binding protein involved in membrane dynamics and stress responses [59,60]. It has been shown to promote radioresistance in NPC by inhibiting PI3K/mTOR and enhancing autophagic activity [61]. Similarly, the lncRNA CASC19, which modulates oncogenic pathways like Wnt/β-catenin and EMT in various cancers [62,63,64], confers radioresistance in NPC by promoting autophagy through the AMPK-mTOR Pathway. Notably, CASC19 knockdown via siRNA suppresses autophagy and enhances apoptosis through PARP1 activation, thereby increasing radiosensitivity [43]. 

Another pivotal mechanism facilitating radioresistance involves autophagy activation through Beclin-1. The lysosome-associated transmembrane protein 4β (LAPTM4B) was identified as a critical regulator that interacts with Beclin-1 to trigger autophagosome formation in epidermal growth factor receptor (EGFR)-overexpressing radioresistant NPC cells [65]. Overexpression of LAPTM4B enhances autophagy through physical association with Beclin-1 and confers radioresistance, whereas its silencing supresses autophagy initiation and sensitises cells to ionising radiation.

Furthermore, EGFR and LAPTM4B were found to interact together and stabilise one another within endosomes [65]. Similarly, Wnt5A-overexpressing NPC cells accumulate significantly more autophagosomes and are more radioresistant, through regulation of Beclin-1 [66].

Selective forms of autophagy, such as mitophagy which recycles damaged mitochondria, have also been implicated in NPC radioresistance. A study by Chen et al. [67] identified β-lactamase-like protein 2 (LACTB2) as a protein highly elevated in radioresistant NPC cells and in the serum of NPC patients postradiotherapy. LACTB2 localises to mitochondria and interacts with the *N*-terminal domain of PINK1 to provoke PINK1/Parkin-dependent mitophagy in irradiated NPC cells. This process facilitates the removal of dysfunctional mitochondria, a major source of reactive oxygen species (ROS); therefore, LACTB2 provides a survival advantage to irradiated NPC cells. LACTB2 inhibition sensitised NPC cells to radiation. This suggests that radioresistant NPC cells may rely on mitophagy as a specialised pathway to mitigate mitochondrial DNA damage and oxidative stress inflicted by radiation. 

In contrast, various studies demonstrate autophagy may enhance radiosensitivity in NPC. A genome-wide CRISPR screen highlighted the RNA splicing factor LUC7L2 as a novel driver of autophagy-mediated NPC radioresistance [68]. LUC7L2 was found to associate with and maintain the expression of the autophagy receptor SQSTM1/p62. Knockdown of LUC7L2 led to a reduction in p62 levels and an increase in autophagic activity. The loss of LUC7L2 not only impaired cell proliferation and clonogenic survival after radiation but also synergised with the autophagy inhibitor chloroquine to induce even greater cell death. High LUC7L2 expression also correlated with advanced tumour stage and poorer overall survival (OS). However, this study does not elucidate the precise molecular mechanism linking increased autophagy to radiosensitivity. 

Lin et al. [69] demonstrated that protein tyrosine phosphatase receptor type D (PTPRD) enhances autophagy by dephosphorylating STAT3, resulting in enhanced transcription of ATG5. This was evidenced by elevated LC3-II/LC3-I ratios and decreased p62 levels. Immunohistochemical staining of NPC tissues further revealed that low PTPRD and high phosphorylated-STAT3 (p-STAT3) levels were associated with an advanced stage, increased mortality, distant metastasis, and recurrence [69]. Another example where elevated autophagy is associated with radiosensitivity is kinesin family member 15 (KIF15), where its knockdown results in accumulation of autophagosomes and stimulation of the STAT3/ATG7 pathway, thereby sensitising NPC cells and xenograft models to radiation [70]. It is noteworthy that KIF15 is stabilised by METTL3 [70], which also regulates the expression of the autophagy-associated lncRNAs LINC00313 and ZFAS1 [54,55]. 

### 3.2. Chemoresistance

Autophagy has emerged as a contributor to chemoresistance by enabling tumour cells to survive under physiologically stressful conditions [71]. Zhang et al. [72] identified HS-1-associated protein X-1 (HAX-1) as a mediator of cisplatin resistance in NPC through its regulation of autophagic flux. They demonstrated that HAX-1 overexpression disrupts late-stage autophagy by competitively binding to Rab7a, a GTPase required for autophagosome–lysosomal fusion. This blockade results in the accumulation of SQSTM1/p62 which promotes tumour survival under cisplatin-induced stress. Furthermore, HAX-1 is stabilised by IGF2BP1, creating a positive feedback loop that amplifies autophagy inhibition and chemoresistance. Clinically, elevated HAX-1 levels correlate with reduced OS and progression-free survival (PFS). Functional assays revealed that genetic and pharmacological suppression of HAX-1 restored autophagic flux, reduced SQSTM1 accumulation, and sensitised NPC cells to cisplatin in vitro and in xenograft models [52]. 

These findings highlight the potential utility of HAX-1 as both a prognostic biomarker and a therapeutic target in NPC. Furthermore, CENPN, which recognises the *N*-terminal region of the CENPA nucleosome and binds to CENPL to form a mitophagy-associated network [73], has also been shown to supress autophagy and increase paclitaxel resistance in NPC by inhibiting the CREB-VAMP8 signalling axis. In nude mice, knockdown of CENPN promoted autophagy and augmented the sensitivity of NPC to paclitaxel. Long-term survival analysis of 45 NPC patients with 10-year follow-up revealed that high CENPN expression significantly predicted poor prognosis [74]. 

In contrast to its discussed role in radioresistance, Parkin has interestingly been shown to enhance sensitivity of NPC to paclitaxel by activating BNIP3/NIX-mediated mitophagy. Overexpression of Parkin has also been demonstrated to increase ROS, mitochondrial membrane potential, and LC3II/LC3I in paclitaxel-treated C666-1 cells [75]. The discrepancy between these findings and those related to LACTB2-mediated mitophagy may stem from differences in the extent of mitophagy activation, stress context, or experimental design. In the paclitaxel study, Parkin overexpression appeared to exacerbate mitochondrial dysfunction and promote excessive mitophagy, leading to elevated ROS levels and enhanced apoptosis in NPC cells. In contrast, LACTB2-mediated activation of the PINK1/Parkin pathway may be associated with more controlled mitophagy which leads to a reduction in intracellular ROS levels. This may suggest that moderate levels of mitophagy, as seen during radiotherapy-induced stress, selectively remove damaged mitochondria, thereby supporting mitochondrial health and reducing oxidative stress. Conversely, under chemotherapeutic stress, Parkin overexpression may push mitophagy beyond a protective threshold, resulting in mitochondrial collapse and oxidative injury. It is also notable that in the paclitaxel study, Parkin was artificially overexpressed, which may not fully represent physiological conditions. Nevertheless, comparative mechanistic studies are needed to conclusively determine the mechanisms underlying the dual role of Parkin in mitochondrial function and therapeutic resistance. 

**Figure 4:**
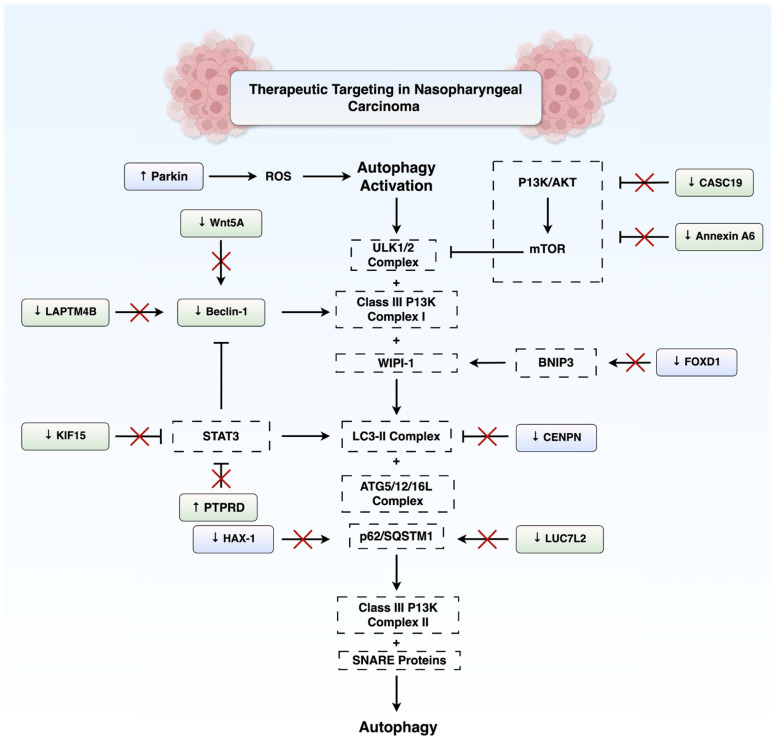
**Potential therapeutic targets regulating autophagy in NPC.** This schematic summarises molecular targets within the autophagy signalling pathway that could enhance therapeutic outcomes in NPC. Green boxes denote targets associated with radiosensitisation, while purple boxes represent targets associated with chemosensitisation. Abbreviations: AKT, protein kinase B; ATG, autophagy-related gene; Beclin-1, BCL2-interacting protein; BNIP3, BCL2-interacting protein 3; CASC19, cancer susceptibility candidate 19; CENPN, centromere protein N; FOXD1, forkhead box D1; HAX-1, HCLS1-associated protein X-1; KIF15, kinesin family member 15; LAPTM4B, lysosomal protein transmembrane 4 beta; LC3, microtubule-associated protein 1A/1B-light chain 3; LUC7L2, LUC7-like 2 pre-mRNA splicing factor; mTOR, mammalian target of rapamycin; NPC, nasopharyngeal carcinoma; PIK3, phosphoinositide 3-kinase; PTPRD, protein tyrosine phosphatase receptor type D; ROS, reactive oxygen species; SNARE, soluble NSF attachment protein receptor; SQSTM1/p62, sequestosome 1; STAT3, signal transducer and activator of transcription 3; ULK1/2, Unc-51-like autophagy activating kinase 1/2; WIPI-1, WD repeat domain phosphoinositide-interacting protein 1. Figure created using BioRender.com (URL accessed on 31 July 2025) and Microsoft PowerPoint.

FOXD1, a member of the forkhead box family of transcription factors, has also been implicated in chemoresistance. FOXD1 is significantly overexpressed in NPC and correlates with poor OS [76]. Mechanistically, FOXD1 promotes NPC cell proliferation, migration, invasion, and gemcitabine resistance by transcriptionally activating BNIP3. Notably, the stability and nuclear localisation of FOXD1 are potentiated by *N*-glycosylation at Asn176 and mediated by ALG3, which directly interacts with FOXD1 [76]. As such, targeting the ALG3-FOXD1-BNIP3 axis may represent a promising therapeutic approach to overcome chemoresistance and inhibit tumour progression. Figure 4 summarises the key autophagy-related signalling pathways and potential molecular targets implicated in both radioresistance and chemoresistance in NPC.

An emerging and intriguing mechanism of chemotherapy resistance in NPC involves the autophagy-driven formation of dormant polyploid giant cancer cells (PGCCs). Chemotherapy results in the activation of autophagy via ATP depletion which activates the AMPK/mTOR pathway to promote the formation of PGCCs [77]. The scaffolding function of RIPK1 is necessary for PGCC survival. PGCCs are a dormant cell population strongly associated with advanced stage, short time to recurrence, metastasis, and poor OS [77]. Importantly, autophagy inhibition with drugs such as hydroxychloroquine (HCQ) or BML-275 prior to chemotherapy prevented PGCC formation and significantly reduced metastasis and improved survival in murine models [77]. These findings suggest that assessing PGCC levels at diagnosis may help stratify high-risk NPC patients likely to benefit from adjuvant autophagy inhibition.

### 3.3. Resistance to Immunotherapy

Recent evidence uncovered a novel mechanism of immune evasion in NPC, whereby galectin-9 (G9) interacts with cytotoxic T lymphocytes (CTLs) to induce autophagy and promote resistance to T cell-mediated killing [78]. Cell surface G9 enhances CTL–tumour cell contact without causing CTL dysfunction, instead shifting tumour cell fate towards autophagy rather than necrosis. This autophagy suppresses necrotic death, impairs granzyme B uptake, and reduces CTL cytotoxic efficacy. Pharmacological inhibition of autophagy restored tumour cell sensitivity to CTL-mediated killing. Clinically, high G9 expression correlated with increased autophagy markers, reduced necrosis, and poorer survival outcomes in NPC patients [78]. These findings suggest that G9-induced autophagy may also attenuate the response to immune checkpoint inhibitors (ICIs), warranting further investigation. 

## 4. Autophagy-Related Proteins as NPC Biomarkers and Predictors of Therapeutic Resistance

As outlined throughout this review, several autophagy-related proteins (CENPN, PTPRD, SQSTM1, TIPE1, WIPI1) implicated in NPC pathogenesis and therapeutic response also carry prognostic significance. In addition to these, other autophagy-related genes have been studied more directly as clinical biomarkers predictive of prognosis and treatment outcomes in NPC. Table 1 summarises several autophagy-related proteins that have been proposed as prognostic biomarkers in NPC. While all listed markers, including TIPE1, WIPI1, PTPRD, and CENPN, demonstrate mechanistic relevance and potential clinical significance, we have chosen to subsequently further elaborate on the role of SQSTM1, Beclin-1, and AURKA as potential biomarkers due to the availability of more comprehensive, standalone studies specifically focused on their prognostic value. 

### 4.1. SQSTM1/p62

The multifunctional autophagy adaptor protein SQSTM1/p62 has been implicated in the metastatic progression of high-risk NPC, particularly in patients with T4 or N2-3 stage disease. In a comprehensive study, Yang and colleagues [44] demonstrated through both in vitro and in vivo experiments that SQSTM1 overexpression promotes EMT via activation of the NF-κB signalling pathway. Notably, inhibition of SQSTM1 was shown to sensitise cells to cisplatin-induced apoptosis. Immunohistochemical analysis of 116 NPC samples revealed that high SQSTM1 expression was significantly associated with reduced distant metastasis-free survival (DMFS), PFS, and OS. Based on these findings, a prognostic model combining SQSTM1 expression and N-stage was developed to stratify patients by risk of distant metastasis. To validate the “SN model”, a separate cohort of 134 patients from a prospective clinical trial was examined comparing CCRT alone versus induction chemotherapy plus CCRT. High-risk patients (advanced N-stage and high SQSTM1) benefitted significantly from the addition of induction chemotherapy, while low- and intermediate-risk patients did not. These findings underscore the clinical utility of SQSTM1 as both a prognostic biomarker and a predictive tool for treatment stratification. 

### 4.2. Beclin-1

Beclin-1, a core component of the autophagy initiation complex, has also emerged as a clinically relevant biomarker in NPC. In a randomised cohort of 128 patients treated with induction chemotherapy plus radiotherapy or chemoradiotherapy, elevated Beclin-1 expression was significantly associated with poorer OS, PFS, and DMFS, though not with local failure-free survival [80]. Notably, Beclin-1 expression showed a positive correlation with hypoxia-inducible factor 1-alpha (HIF-1α), a hypoxia-inducible transcription factor known to promote tumour aggressiveness. Among patients with high HIF-1α expression, those with lower Beclin-1 levels exhibited significantly improved survival (*p* = 0.036), suggesting that hypoxia-associated autophagy may promote resistance to chemoradiotherapy [80]. These findings suggest Beclin-1 as a negative prognostic marker in NPC, particularly relevant in the context of tumour hypoxia.

### 4.3. Aurora Kinase A

Aurora kinase A (AURKA), a serine/threonine kinase involved in mitogenic progression, has recently been implicated as a potential autophagy-associated biomarker in NPC. In one study, transcriptomic analyses and validation in clinical tissue samples revealed that AURKA is significantly overexpressed in NPC compared to nasopharyngitis [79]. In addition, elevated AURKA expression correlated with poorer clinical outcomes. Functional assays demonstrated that silencing AURKA in NPC cell lines inhibited both proliferation and migration. Gene expression analysis stratified by AURKA levels showed differential enrichment of autophagy-related pathways, including the mTOR and p53 axes, suggesting AURKA may act upstream of autophagic regulation. Notably, AURKA expression was inversely correlated with positive autophagy regulators such as MAP1LC3B and directly associated with known autophagy suppressors like BIRC5 and ATIC. Interestingly, AURKA was found to negatively correlate with immune infiltration, including CD8+ T cells, suggesting an additional role in modulating the tumour immune microenvironment [79]. Together, this highlights AURKA as a multifaceted biomarker with relevance to both autophagy regulation and immune evasion in NPC.

## 5. Therapeutic Targeting of Autophagy in NPC

### 5.1. Autophagy Activation

Therapeutic strategies that promote autophagy are under investigation in NPC. Taurine (2-aminoethane-sulfonic acid), a naturally occurring sulfonic acid lacking a carboxyl group and therefore not participating in protein synthesis, has been shown to co-activate autophagy and apoptosis in vitro and in xenograft models [81]. Immunohistochemical analysis in one study revealed increased expression of cleaved caspase-3, p53, LC3B, and Beclin-1 in taurine-treated tumours. These effects are potentially attributable to taurine’s upregulation of TFEB and downregulation of ERK1/2 signalling. The volume and weight of the tumours were also significantly lower in the taurine-treated group in comparison with in the distilled-water-treated control group [81].

Oncolytic virus therapy represents another promising approach to trigger autophagy-dependent death through selective replication within tumour cells. Huang et al. [82] demonstrated that the Orf parapoxvirus (ORFV) potently induces autophagy in NPC cells by inhibiting the PI3K/AKT/mTOR pathway. ORFV-treated NPC cells showed a rise in LC3-II levels, a drop in p62, an upregulation of autophagy-related gene, and increased autophagosomes. Blocking autophagy with chloroquine or 3-methyladenine partially rescued NPC cells from virus-induced cytotoxicity, demonstrating that ORFV exerts anti-tumour effects through autophagy-driven apoptosis [82]. 

A related strategy involves the use of lncRNAs that modulate autophagy. Xu et al. [83] demonstrated that the circle RNA circLASP1 acts as an upregulated oncogenic autophagy suppressor in NPC. circLASP1 functions as a molecular sponge for miR-625, which normally inhibits AKT/mTOR signalling. This was shown to contribute to NPC cell proliferation and resistance to the AKT inhibitor MK-2206. Silencing of circLASP1 also heightened autophagy and apoptosis and dramatically sensitised NPC to treatment with MK-2206 treatment [83]. Collectively, these studies highlight that the enhancement of autophagy beyond a physiological threshold towards apoptosis may be a potential therapeutic avenue in NPC.

### 5.2. Autophagy Inhibition

Given the dual role of autophagy in tumour biology, inhibition of autophagy may also yield therapeutic benefits. A novel mechanism is through the targeting of chromatin-modifying enzymes such as euchromatic histone-lysine *N*-methyltransferase 2 (EHMT2) or G9a, a histone H3K9 methyltransferase overexpressed in NPC [84]. Inhibition of G9a using siRNA or the small molecule BIX-01294 significantly impaired autophagic flux in NPC and reduced cell proliferation. Interestingly, BIX-01294 initiated autophagosome formation in a Beclin1-independent manner but then inhibited lysosomal cathepsin D, preventing autophagolysosomal function. This disruption led to accumulation of dysfunctional autophagic vacuoles and caspase-independent cell death, offering a unique mechanism of tumour suppression [84]. 

### 5.3. Targeting Autophagy Using Natural Compounds 

The use of natural compounds as a therapeutic strategy in NPC has generated attention, particularly in East Asian studies, due to their potential efficacy and low toxicity profiles. Current evidence remains limited to preclinical and animal models; nevertheless, several compounds have demonstrated the ability to modulate autophagy alongside other anti-tumour effects. A notable example is berberine, an isoquinoline alkaloid derived from *Coptis chinensis*. Berberine was originally used as a broad-spectrum antibacterial agent [85]. It has been shown to exert antitumoural effects in various cancers such as colorectal cancer, lung cancer, and NPC [85]. Specifically, it inhibits the activation of STAT3 induced by tumour-associated fibroblasts to suppress the growth of NPC in the C666-1 cell line [86]. Wu et al. [87] also demonstrated that berberine induces autophagy in NPC by enhancing EGFR transcription leading to activation of RAS-RAF-MEK-ERK signalling. Interestingly, BBR was found to activate a specific super-enhancer (SE) in NPC cells that was absent in untreated cells, proposed to drive the expression of EGFR. Treatment with berberine was also shown to inhibit proliferation, metastasis, and invasion, underscoring its therapeutic potential.

Curcumol, a guaiane-type sesquiterpenoid derived from *Curcuma* species, has also shown anti-NPC activity. It has been demonstrated to inhibit the invasion and migration of the same cell line by targeting nucleolin to inhibit VEGFA/VEGFR1/PI3k/AKT signalling [88]. Curcumol treatment downregulates the expression of nucleolin, a multifunctional RNA-binding protein, by interacting with its RNA-binding domain 2 [89]. This attenuates PI3k/Akt/mTOR signalling. In curcumol-treated NPC cells of the C666-1 line, levels of phosphorylated Akt, mTOR, and p62 were decreased, and the LC3-II/LC3-I ratio increased, all consistent with enhanced autophagic flux. Curcumol was also found to inhibit the proliferation and growth of these cells [89]. 

Astragaloside IV, a bioactive saponin from *Astragalus membranaceus*, has been shown to inhibit the progression of NPC by downregulating the expression of the transcription factor SATB2 to suppress Wnt signalling [90]. Treatment with AS-IV reduced cell viability, migration, and invasion, while promoting apoptosis and reducing autophagy. In vivo, AS-IV reduced tumour growth and LC3B levels, suggesting reduced autophagic activity and proliferation in NPC xenografts [90]. 

Geraniin, an ellagitannin found in several fruits and nuts, was shown to inhibit the viability of C666-1 cells and induce autophagy-mediated cell death [91]. Geraniin treatment enhanced ROS production and suppressed NF-κB and β-catenin expression. Western blot analysis revealed that geraniin downregulated PI3K/Akt/mTOR signalling while increasing LC3 and ATG7 expression, supporting the activation of autophagy [91]. 

In one study, isogarcinol, isolated from *Garcinia oblongifolia*, exerted anti-NPC effects by inducing mitochondria-mediated autophagic cell death [92]. Treatment with isogarcinol inhibited proliferation, colony formation, migration, and invasion of NPC cells and increased ROS production and mitochondrial dysfunction. Mechanistically, isogarcinol activated mitochondrial apoptosis by increasing the Bax/Bcl-2 ratio and cleaved caspase-3, while promoting LC3-II accumulation and p62 upregulation, suggesting blocked autophagic flux [92]. These effects were confirmed in vivo, where isogarcinol suppressed tumour growth with low toxicity.

Oligonol, a polyphenolic compound derived from lychee, has demonstrated anti-NPC activity through concurrent induction of apoptosis and autophagy [93]. Oligonol treatment reduced viability and increased apoptotic markers, including cleaved caspase-8 and caspase-3, PARP, and cytokeratin-18 fragments. It also upregulated Beclin-1 and LC3-II, consistent with enhanced autophagy. Interestingly, inhibition of autophagy using LY294002 or 3-MA amplified the apoptotic effects of oligonol [93].

Pentagalloylglucose (PGG), a natural polyphenol, was shown to inhibit NPC cell proliferation and migration and induce both apoptosis and autophagy [94]. PGG increased the Bax/Bcl-2 ratio and LC3B levels while reducing cyclin D1 and β-catenin expression. The underlying mechanism involved activation of p38 MAPK and GSK3β, with concurrent downregulation of mTOR and Wnt/β-catenin signalling. In vivo, PGG effectively suppressed tumour growth and lung metastasis, supporting its therapeutic potential against NPC [94].

Qing Yan Li Ge Tang (QYLGT), a traditional Chinese herbal formula, has been demonstrated to suppress NPC cell viability and colony formation by inducing autophagic cell death [95]. QYLGT was shown to activate the PI3K/Akt/mTOR pathway and upregulate Atg3, Atg6, and Atg12-Atg5 conjugate levels. This effect was reversed by autophagy inhibitors, confirming its dependence on autophagy [95].

In one study, theaflavin, a polyphenol from black tea, inhibited NPC cell proliferation and promoted apoptosis through autophagy induction [96]. In CNE2 cells, theaflavin treatment increased LC3 and LAMP1 levels, while reducing p62 expression. This autophagy activation was mediated via inhibition of the mTOR pathway. Blocking autophagy with chloroquine attenuated theaflavin-induced apoptosis, suggesting that the pro-apoptotic effect of theaflavin is dependent on autophagy induction [96].

Trifolirhizin, a flavonoid glycoside, suppressed NPC cell proliferation and promoted apoptosis and autophagy by targeting protein tyrosine kinase 6 (PTK6) [97]. Molecular docking and expression studies confirmed PTK6 as a direct target of trifolirhizin. Treatment reduced PTK6 levels and upregulated autophagy-related proteins, including LC3. Upregulation of PTK6 partly abolished these effects, supporting the conclusion that trifolirhizin induces autophagy and suppresses malignancy via PTK6 inhibition [97]. Table 2 summarises the effects of additional natural compounds on NPC cells, detailing their sources, primary molecular mechanisms, role in modulation of autophagy, and overall impact on cell behaviour.

### 5.4. Barriers to the Clinical Translation of Autophagy Modulators

There are various challenges in the clinical translation of therapies that target autophagy. As discussed in this review, autophagy has a dual and context-dependent role in cancer, and as such a simple inhibition or activation strategy may be ineffective. Clinical trial results for monotherapies have been variable. In a phase I trial [98] using HCQ as a neoadjuvant in early-stage solid tumours, HCQ induced secretion of the tumour suppressor Par-4, correlating with tumour apoptosis in most patients. Inhibition of autophagy, as measured by p62 accumulation, was observed in all treated patients regardless of tumour response. However, a randomised placebo-controlled phase II trial in breast cancer patients using single-agent chloroquine at 500 mg daily did not show significant effects on tumour proliferation compared to a placebo, as measured by Ki67 [99]. In addition, this trial had a notable dropout rate due to mild toxicities [99]. Similarly, a phase II study of HCQ in metastatic pancreatic cancer reported inconsistent pharmacodynamic evidence of autophagy inhibition and minimal clinical efficacy, with only 10% of patients remaining progression-free at two months [100]. These results suggest that the limited efficacy in monotherapy trials may be attributable to inadequate autophagy inhibition, dose-limiting toxicities, or a lack of patient stratification. As autophagy is a systemically required process, its targeting may raise issues surrounding toxicity. For instance, knockout of Atg7 in mice resulted in neurodegeneration, and it increased susceptibility to infection and poor survival [101]. Fortunately, clinical trials so far indicate that autophagy inhibitors like HCQ are reasonably well-tolerated at doses typically ranging from around 200 to 1200 mg/day [102,103,104]. However, toxicities observed have included haematological abnormalities like neutropaenia and gastrointestinal symptoms such as nausea and vomiting [102,103]. A potential advantage in the clinical translation of autophagy-modulating drugs like HCQ and rapamycin, which have been approved for various indications [105,106], is that their safety and dosing parameters are relatively well-defined. For instance, HCQ has been used to treat conditions like malaria, rheumatoid arthritis, and systemic lupus erythematosus [106]. However, its long-term use is associated with retinal toxicity that necessitates frequent ophthalmological review [107]. While the novel drugs discussed in this review show promising pre-clinical results, their safety and efficacy profiles in clinical settings are yet to be elucidated.

## 6. Future Perspectives and Ongoing Research

Although the clinical application of autophagy-targeting agents as monotherapy remains limited, modulating autophagy in combination with existing therapies presents a promising approach to overcome therapeutic resistance in NPC. Platinum-based chemotherapeutics such as cisplatin and its analogue nedaplatin trigger autophagy in NPC cells and tumours, which has been linked to reduced drug sensitivity. Biopsy samples from patients undergoing chemotherapy have demonstrated increased expression of autophagy markers such as Beclin-1 and LC3 [108]. In vitro studies further revealed that combining cisplatin with the autophagy inhibitor chloroquine significantly reduced NPC cell viability and enhanced apoptosis compared to cisplatin monotherapy [108]. Similarly, in cisplatin-resistant NPC cell lines, nedaplatin treatment led to marked autophagosome accumulation and increased LC3-II levels, indicative of enhanced autophagy [109]. Mechanistically, nedaplatin was shown to activate ERK and inhibit Akt/mTOR signalling. Blocking autophagy in these resistant cells using pharmacologic inhibitors like bafilomycin A1 or 3-methyladenine substantially potentiates nedaplatin’s cytotoxicity, restoring apoptotic activity and ROS accumulation [109]. 

Additionally, chloroquine treatment was shown to downregulate expression of key ATP-binding cassette (ABC) transporters, including ABCB1, ABCC1, and P-glycoprotein (P-gp), which are known to confer multidrug resistance through ATP-dependent drug efflux [77,78].

Modulating autophagy has also shown potential in enhancing the radiosensitivity of NPC. Apogossypolone is a small-molecule inhibitor which prevents the binding of Bcl-2 to Bax and Beclin-1, thereby stimulating autophagy. This was evidenced through increased LC3-II expression and decreased p62 levels [110]. It has been shown to enhance the radiosensitivity of NPC cells both in vitro as measured via colony formation assays and in xenograft models. More recently, a novel radiosensitisation approach involved inhibition of the replication protein A (RPA) complex using the small molecule (1Z)-1-[(2-hydroxyanilino)methylidene]naphthalen-2-one (HAMNO) [80]. HAMNO significantly enhanced the response of NPC cells and xenografts to radiation but was also found to potently induce autophagy via AMPK/mTOR activation and transcriptional upregulation of autophagy genes such as WIPI1 and SQSTM1. Notably, when HAMNO was combined with chloroquine or ATG5 knockdown, this further amplified antitumour effects, suggesting that a dual-targeting approach may be particularly efficacious. These studies highlight the complexity of autophagy in radio- and chemosensitisation, suggesting that carefully balancing its activation or inhibition may be key to maximising therapeutic outcomes. Further studies to elucidate the context-dependent role of autophagy in governing therapeutic efficacy may provide a biological foundation for clinical trials in NPC.

The integration of artificial intelligence (AI), particularly machine learning, into oncology research offers novel avenues for elucidating the complex role of autophagy in tumour progression, treatment response, and prognostication. In a recent study by Wang et al. [111], a machine learning-based autophagy-related prognostic signature (ARPS) was developed for bladder cancer using transcriptomic datasets from multiple cohorts. Through evaluation of 101 algorithm combinations, the authors identified the key autophagy-related genes with high predictive value [81]. This ARPS demonstrated superior prognostic accuracy over existing clinical markers, correlated with immune microenvironment differences, and it offered insights into differential responses to immunotherapy. The study further validated FKBP9 as a key oncogenic autophagy-related gene via in vitro and in vivo functional assays. In a complementary study, He et al. employed a supervised learning approach using immunohistochemical data to quantify the autophagy-related proteins, ATG1, ATG5, LC3B, and p62, in renal cell carcinoma (RCC) [82]. A K-nearest neighbour algorithm was trained to distinguish RCC subtypes based on basal autophagy levels. Their approach revealed reduced autophagy in tumours and demonstrated how quantified autophagy markers could aid histopathological classification and prognosis. These studies highlight the potential power of AI-driven approaches in uncovering clinically relevant autophagy signatures and improve classification, risk stratification, and treatment selection across many cancer types. The application of novel research techniques to NPC could accelerate biomarker discovery and facilitate the implementation of enhanced personalised treatment strategies.

## 7. Conclusions

Autophagy represents a multifaceted and dynamic process in the pathogenesis and treatment of NPC. It plays a critical role in regulating tumourigenesis, metastatic potential, immune evasion, and resistance to radiotherapy, chemotherapy, and immunotherapy. Numerous autophagy-related proteins, non-coding RNAs, and signalling pathways have emerged as candidate biomarkers and therapeutic targets, offering new opportunities for precision medicine in NPC. Experimental interventions such as genetic modulation, pharmacological inhibitors, and bioactive natural compounds have demonstrated the capacity to modulate autophagy and reverse therapy resistance in a variety of preclinical models. However, the dualistic nature of autophagy, wherein it can either support cell survival or promote cell death depending on the context, necessitates precise regulation to achieve therapeutic benefit. Future research should focus on elucidating the molecular determinants that govern this context-specificity and on integrating autophagy modulation into combinational treatments. Ultimately, a deeper understanding of autophagy’s dualistic nature, combined with integrative translational research, will be essential for harnessing its full therapeutic potential in improving outcomes for NPC patients.

## Figures and Tables

**Table 1:** Autophagy-related prognostic biomarkers in NPC.

Biomarker	Autophagy-Regulating Mechanism	Prognostic Impact	Therapeutic Resistance	Patient Cohort Details	Detection Method	Ref.
AURKA	Activates mTOR/ULK1 pathway, thereby suppressing autophagy; linked to reduced immune infiltration and poor immune landscape in high-AURKA tumours.	High expression associated with poorer OS and DMFS	Not directly evaluated in study.	*n* = 208 NPC patients; validation in TCGA data	IHC and qRT-PCR	[79]
Beclin-1	Core autophagy initiator protein. High Beclin-1 coexpresses with HIF-1α, possibly promoting survival under hypoxia and autophagy-mediated therapeutic resistance.	High expression associated with poorer OS, PFS, and DMFS	High Beclin-1 with high HIF-1α correlates with resistance to chemoradiotherapy.	*n* = 128 advanced NPC patients (RCT-derived); divided into training (*n* = 61) and testing set (*n* = 67)	IHC	[80]
CENPN	Suppresses autophagy by inhibiting the CREB-VAMP8 signalling axis, reducing autophagosome formation and increasing paclitaxel resistance.	High expression associated with poorer OS and DFS	Not directly evaluated in study.	*n* = 98 NPC patients; stages I–IV	IHC, Western blot, qRT-PCR	[74]
PTPRD	Promotes radiation-induced autophagy via STAT3 dephosphorylation → increased ATG5 transcription → autophagic flux enhancement.	Low expression associated with poorer OS	Low PTPRD associated with radioresistance; overexpression enhances radiosensitivity via STAT3 dephosphorylation leading to increased ATG5 and autophagy.	*n* = 107 NPC patient samples	IHC, qRT-PCR, bisulfite pyrosequencing	[69]
SQSTM1	Downstream of impaired autophagic flux. Its accumulation activates NF-κB and induces EMT, linking defective autophagy to metastasis.	High expression associated with increased risk of distant metastasis	Inhibiting SQSTM1 enhanced sensitivity to cisplatin in NPC cells.	*n* = 116 NPC patients (retrospective); validation cohort *n* = 134 (prospective RCT)	IHC, Western blot, qRT-PCR	[44]
TIPE1	Inhibits autophagy by blocking AMPK activation and enhancing mTOR signalling → leads to reduced LC3-II and increased p62 accumulation.	High expression associated with poorer OS	Not directly evaluated in study.	*n* = 108 NPC patients	IHC, qRT-PCR, Western blot	[41]
WIPI1	Enhances starvation-induced autophagy via TRIM21 interaction; loss of WIPI1 decreases autophagic activity and may promote Myc-driven proliferation.	Low expression associated with poorer PFS	Not directly evaluated in study.	*n* = 17 NPC tissues vs. 14 normal tissues; in vitro and in vivo models	qRT-PCR, Western blot, RNA-seq	[38]

Abbreviations: AKT, protein kinase B; AMPK, AMP-activated protein kinase; ATG5, autophagy-related gene 5; CENPN, centromere protein N; CREB, cAMP response element-binding protein; DMFS, distant metastasis-free survival; EMT, epithelial-to-mesenchymal transition; HIF-1α, hypoxia-inducible factor 1-alpha; IHC, immunohistochemistry; LC3-II, microtubule-associated protein 1A/1B-light chain 3-II; mTOR, mechanistic target of rapamycin; NF-κB, nuclear factor kappa-light-chain-enhancer of activated B cells; NPC, nasopharyngeal carcinoma; OS, overall survival; PFS, progression-free survival; PTPRD, protein tyrosine phosphatase receptor type D; qRT-PCR, quantitative reverse transcription polymerase chain reaction; RCT, randomised controlled trial; RNA-seq, RNA sequencing; SQSTM1/p62, sequestosome 1; STAT3, signal transducer and activator of transcription 3; TIPE1, tumour necrosis factor-α-induced protein 8-like 1; TRIM21, tripartite motif-containing 21; ULK1, Unc-51-like autophagy activating kinase 1; VAMP8, vesicle-associated membrane protein 8; WIPI1, WD repeat domain phosphoinositide-interacting protein 1.

**Table 2:** An overview of the various natural compounds shown to modulate autophagy in NPC.

Compound	Source of Compound	Experimental Model	Primary Mechanism	Effect on Autophagy	Effect on NPC Cells	References
**Berberine (BBR)**	*Coptis chinensis*	NPC cells (S18, 5-8F, C666-1)	Activates EGFR → RAS/RAF/MEK/ERK; SE-driven autophagy.	Induces autophagy	Inhibits proliferation, migration, invasion.	[87]
**Curcumol**	Curcuma species	NPC C666-1 cells; xenografts	Targets nucleolin; inhibits PI3K/Akt/mTOR.	Induces autophagy	Suppresses proliferation.	[89]
**Isogarcinol**	*Garcinia oblongifolia*	NPC CNE1/CNE2; xenografts	Autophagy-regulating mechanism not fully elucidated.	Induces autophagy, blocks autophagic flux	Blocks proliferation, migration, invasion; induces mitochondrial apoptosis	[92]
**Astragaloside IV (AS-IV)**	*Astragalus membranaceus*	NPC C666-1/HK-1; xenografts	Suppresses SATB2/Wnt signalling.	Inhibits autophagy	Inhibits proliferation, migration; enhances apoptosis.	[90]
**Pentagalloylglucose (PGG)**	*Paeonia lactiflora*, Galla Rhois	NPC CNE1/CNE2 cells; xenografts	Upregulates p38 MAPK; downregulates Wnt/β-catenin and mTOR.	Induces autophagy	Reduces proliferation, migration; triggers apoptosis and autophagy.	[94]
**Oligonol**	Lychee fruit polyphenol	NPC TW01, HK1 cells	Autophagy-regulating mechanism not fully elucidated.	Induces autophagy	Decreases cell viability.	[93]
**Qing Yan Li Ge Tang**	Chinese herbal formula	NPC TW01 cells	Activates autophagy via PI3K/Akt/mTOR pathway.	Induces autophagy	Inhibits proliferation and colony formation; triggers autophagy-mediated cell death.	[95]
**Theaflavin**	Black tea (*Camellia sinensis*)	NPC CNE2 cells	Inhibits mTOR.	Induces autophagy	Suppresses proliferation; promotes apoptosis.	[96]
**Geraniin**	Phyllanthus species	NPC C666-1 cells	Modulates PI3K/Akt/mTOR.	Induces autophagy	Inhibits proliferation; enhances ROS generation.	[91]
**Trifolirhizin**	*Sophora flavescens*	NPC C666-1 cells	Targets PTK6.	Induces autophagy	Reduces proliferation; promotes apoptosis.	[97]
